# Supplementary material for: Causal Relationship between Aging and Anorexia Nervosa: A White-Matter-Microstructure-Mediated Mendelian Randomization Analysis
Source: Biomedicines. 2024 Aug 16;12(8):1874. doi: 10.3390/biomedicines12081874 (PMC11351342; doi:10.3390/biomedicines12081874)
Supplement: Supplementary file 1 [file biomedicines-12-01874-s001.zip › Supplementary Figure/Figure S4/Singe SNP plot of MR analysis for TL to ubm-b-1751.pdf]

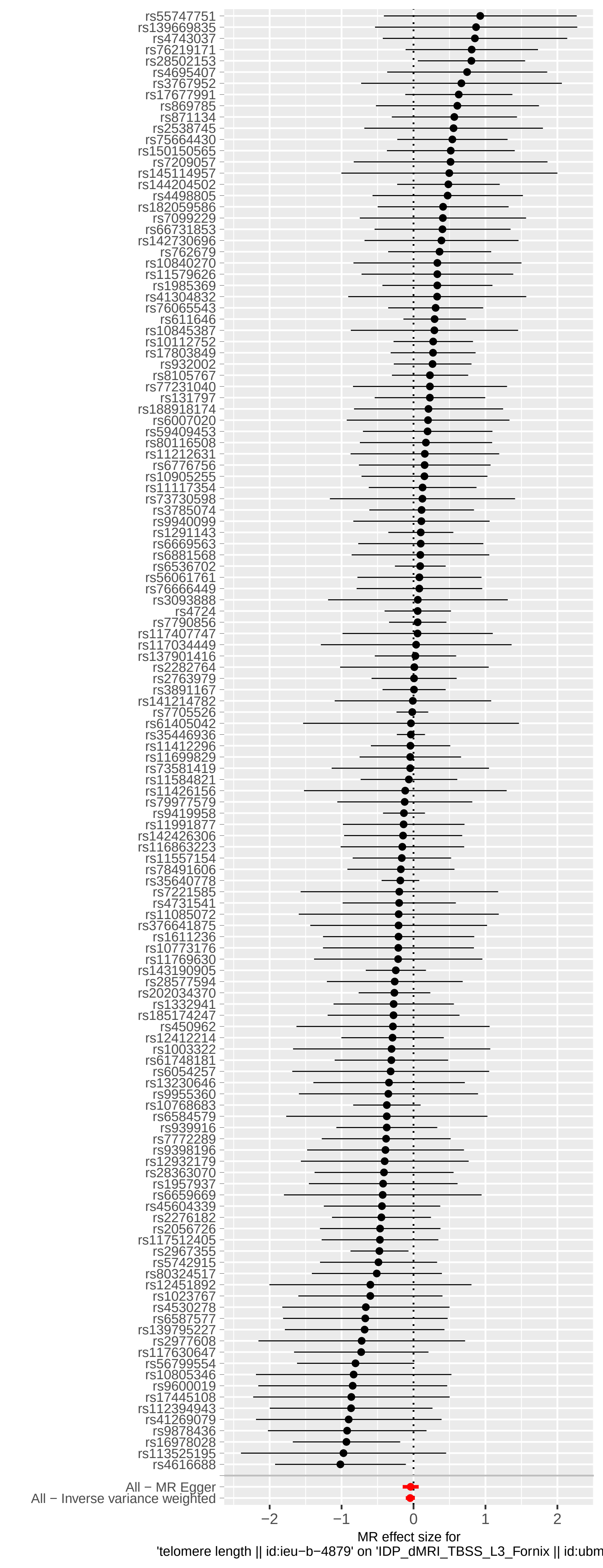
rs55747751
rs139669835
rs4743037
rs76219171
rs28502153
rs4695407
rs3767952
rs17677991
rs869785
rs871134
rs2538745
rs75664430
rs150150565
rs7209057
rs145114957
rs144204502
rs4498805
rs182059586
rs7099229
rs66731853
rs142730696
rs762679
rs10840270
rs11579626
rs1985369
rs41304832
rs76065543
rs611646
rs10845387
rs10112752
rs17803849
rs932002
rs8105767
rs77231040
rs131797
rs188918174
rs6007020
rs59409453
rs80116508
rs11212631
rs6776756
rs10905255
rs11117354
rs73730598
rs3785074
rs9940099
rs1291143
rs6669563
rs6881568
rs6536702
rs56061761
rs76666449
rs3093888
rs4724
rs7790856
rs117407747
rs117034449
rs137901416
rs2282764
rs2763979
rs3891167
rs141214782
rs7705526
rs61405042
rs35446936
rs11412296
rs11699829
rs73581419
rs11584821
rs11426156
rs79977579
rs9419958
rs11991877
rs142426306
rs116863223
rs11557154
rs78491606
rs35640778
rs7221585
rs4731541
rs11085072
rs376641875
rs1611236
rs10773176
rs11769630
rs143190905
rs28577594
rs202034370
rs1332941
rs185174247
rs450962
rs12412214
rs1003322
rs61748181
rs6054257
rs13230646
rs9955360
rs10768683
rs6584579
rs939916
rs7772289
rs9398196
rs12932179
rs28363070
rs1957937
rs6659669
rs45604339
rs2276182
rs2056726
rs117512405
rs2967355
rs5742915
rs80324517
rs12451892
rs1023767
rs4530278
rs6587577
rs139795227
rs2977608
rs117630647
rs56799554
rs10805346
rs9600019
rs17445108
rs112394943
rs41269079
rs9878436
rs16978028
rs113525195
rs4616688
All − MR Egger
All − Inverse variance weighted
−2
−1
0
1
2
MR effect size for
'telomere length || id:ieu−b−4879' on 'IDP_dMRI_TBSS_L3_Fornix || id:ubm
